# Functional Effects of BoNT-A Application in Masseter Muscle in Patients with Symptoms of Bruxism

**DOI:** 10.3390/toxins17110540

**Published:** 2025-10-31

**Authors:** Krystian Matusz, Artur Drużdż, Natalie Górna, Mariusz Glapiński, Małgorzata Gałczyńska-Rusin, Agata Czajka-Jakubowska, Michał Michalak, Agnieszka Przystańska

**Affiliations:** 1 Department of Anatomy, Poznań University of Medical Sciences, 60-781 Poznań, Poland; 2 Department of Neurology, Municipal Hospital, 60-834 Poznań, Poland; 3 Department of Orthodontics and Temporomandibular Disorders, Poznań University of Medical Sciences, 60-781 Poznań, Poland; 4 Department of Computer Science and Statistics, Poznań University of Medical Sciences, 61-806 Poznań, Poland

**Keywords:** incobotulinumtoxinA, masticatory muscles

## Abstract

Bruxism, defined as a repetitive jaw-muscle activity characterized by clenching or grinding of teeth and/or by bracing or thrusting of the mandible, is a prevalent behavior affecting up to 22% of adults worldwide. While traditionally viewed as a disorder, current understanding recognizes bruxism as a behavior that may have both positive and negative consequences. Objective assessment methods for evaluating the effectiveness of interventions in symptomatic patients remain limited. This article presents the first longitudinal study using myotonometry to quantify changes in masseter muscle following botulinum toxin type A (BoNT-A) treatment in patients with symptoms of bruxism. In total, 57 patients were recruited and their masseter muscle tone, stiffness, elasticity, relaxation time, and creep parameters were measured. Measurements were performed at baseline, 3 weeks, and 3 months post-injection during both rest and maximum voluntary contraction. BoNT-A treatment produced significant improvements in all biomechanical parameters, with the greatest effects observed in patients with the highest baseline muscle values. The objective biomechanical changes correlated with the duration of BoNT-A’s therapeutic effects. These findings establish myotonometry as a valuable tool for objective assessment of masticatory muscle function and demonstrate that BoNT-A produces measurable, long-lasting biomechanical changes in masseter muscle parameters, supporting its possible clinical application in this challenging condition.

## 1. Introduction

The newest meta-analysis and prevalence studies inform that bruxism occurs in up to 22% of the world population [1] and is most commonly observed in individuals under 40 years of age, more frequently in women and those living in large urban areas [2,3]. This means that if the entire global population were considered, bruxism could affect up to 1.8 billion people.

Contemporary understanding of bruxism has evolved significantly from earlier conceptualizations. According to the current international consensus, bruxism is defined as repetitive masticatory muscle activity such as clenching or grinding of teeth and/or by bracing or thrusting of the mandible, which may occur either during wakefulness (awake bruxism, AB) or during sleep (sleep bruxism, SB) [4]. The prevailing consensus holds that SB and AB are not disorders in otherwise healthy individuals but rather behaviors that may serve both positive and negative functions [4,5].

Although bruxism has been classified into distinct types and is widespread in the general population, its etiology remains not fully understood and is considered multifactorial. The multifaceted nature of bruxism reflects complex interactions between central nervous system regulation, psychological factors, and peripheral influences [6], as well as environmental and dental factors. Recent research emphasizes that emotional stress may serve as an intermediate link between various musculoskeletal symptoms, with emotional tension manifesting as muscle tension and potentially leading to symptoms through muscle fatigue and articular tissue breakdown [7]. Other research indicates a hereditary susceptibility to SB, with reports of familial occurrence in 21–50% of patients [8,9]. This is supported by the findings of Rintakoski et al. [10], who identified a sex-independent genetic component more frequently present in SB variants than in other temporomandibular joint (TMJ) disorders. While heredity is acknowledged, its expression appears to be modulated by age and environment, underscoring the incomplete understanding of the underlying mechanisms. Suggestions by Przystańska et al. further bridge the gap between genetics and psychology, indicating a potential association between polymorphic variants of stress-related genes and AB, although this requires further investigation [11]. Stress remains a key triggering and exacerbating factor for bruxism [12]. Emotional load is often transferred to muscle tension, which may lead to symptoms due to muscle fatigue and tissue breakdown. As Manfredi et al. noticed, the role of muscle bracing during wakefulness appears particularly critical to symptom onset, with emotional tension manifesting as increased masticatory muscle activity [13,14]. This psychophysiological pathway represents a key target for therapeutic intervention.

The COVID-19 pandemic vividly highlighted this correlation, leading to a surge in bruxism episodes and related internet searches. This increase, particularly among young adults, underscores the age-related epidemiological variability and the need for targeted preventive strategies [15,16,17]. Although malocclusion is no longer perceived as the sole cause, it may act as a contributing or exacerbating factor, influencing the manifestation of symptoms in predisposed or stressed individuals [18].

While bruxism behavior itself may not directly cause pathology, it can become associated with various negative consequences in some individuals. These may include masticatory muscle symptoms (pain, fatigue, hypertrophy), dental wear, and temporomandibular joint symptoms. However, it is important to note that the relationship between bruxism and these consequences is not always linear or causal, and many individuals with bruxism behavior remain asymptomatic throughout their lives [19].

The decision to treat bruxism should be based on the presence of negative consequences rather than the behavior itself. Current understanding positions bruxism as a “medical gateway for dentistry,” emphasizing the importance of proper patient selection and evidence-based treatment approaches [20]. Treatment should focus on managing symptoms and consequences. Various management options for symptomatic bruxism include occlusal splint therapy, behavioral therapy, transcutaneous electrical stimulation, trigger point injections, ultrasound therapy, acupuncture, biofeedback, low-energy laser therapy, tricyclic antidepressants, and muscle relaxants [21,22,23,24]. Many consider splint therapy the golden standard. It affects occlusion and modifies the peripheral sensory response of masticatory muscles, reducing pressure in the TMJ. Although occlusal splints are usually the first choice for SB management, other methods are becoming more common [25].

Over the past decade, one of the emerging approaches to managing the symptoms of bruxism has been the use of botulinum toxin. This concept originates from the well-established efficacy of botulinum toxin (BoNT) therapy in various neuromuscular disorders, including dystonia, spasticity, and orofacial nerupathic pain, where its beneficial effects on symptom control and health-related quality of life (HRQL) are beyond dispute [26,27]. The translation of these neurological treatment experiences to the field of bruxism management appears both logical and promising. Botulinum toxin application in numerous neuromuscular conditions has gained substantial scientific and clinical traction in recent years. BoNT can decrease pain levels and the frequency of bruxism occurrence and improve patients’ subjective well-being. It thus protects against negative consequences of bruxism such as tooth tissue damage, masticatory muscle hypertrophy and microtrauma, cheek maceration, tension headaches, etc. [28,29,30].

There are seven BoNT serotypes. Due to its large safety margin [31] and long duration of action compared to other serotypes [32], type A (BoNT-A) is the most commonly chosen. BoNT-A is a neurotoxin produced by the Gram-positive anaerobic bacteria Clostridium botulinum. It reversibly inhibits acetylcholine release from presynaptic nerve terminals at neuromuscular junctions [32,33]. It has also been observed that BoNT-A reduces the release of inflammatory mediators, substance P, and glutamate [34,35].

Clinical studies on BoNT effects in primates began in the late 1960s [36]. The first clinical applications of BoNT-A occurred in the early 1970s for selective weakening of extraocular muscles in strabismus treatment. The study’s success led to similar applications in treating blepharospasm and hemifacial spasm [37]. In 1989, the U.S. Food and Drug Administration (FDA) approved its use for treating facial spasmodic disorders, and in the same year, Clark & Berris first used BoNT for aesthetic improvement of facial asymmetry [38]. The first proposal for its use in reduction in bruxism occurrences emerged in 2000 [39]. Since then, numerous studies have been conducted determining doses and suggesting the number of injection points [40,41]. In this context, Prof. Wolfgang Jost’s Pictorial Atlas of Botulinum Toxin Injection: Dosage, Localization, Applications is widely recognized as an authoritative reference. The author designates and describes injection sites in his atlas, providing dose ranges per injection point, ranging from 2 to 3 injections in doses of 5–15 Allergan units (u) per point [42].

Given the complex nature of bruxism and the importance of evidence-based treatment approaches, there is a critical need for objective assessment methods to evaluate the efficacy of therapeutic interventions. Current assessment tools, such as the BruxScreen questionnaire and clinical examination, provide valuable clinical insights but lack the objectivity required for quantitative monitoring [43].

This longitudinal study addresses this gap by employing myotonometry to objectively assess the biomechanical effects of BoNT-A treatment on masticatory muscle function in patients with symptomatic bruxism-related muscle dysfunction. The primary objective is to determine whether BoNT-A induces measurable changes in muscle biomechanical properties that could serve as objective markers of treatment efficacy. This approach emphasizes managing the consequences of problematic bruxism rather than attempting to eliminate the behavior entirely.

The masseter muscle was selected for this study because it elevates the mandible and contributes to lateral and protrusive movements essential for chewing and parafunctional activities [44]. It is also one of the primary muscles activated during bruxism episodes. Being superficially located, the muscle is also easily accessible for examination.

Given the evolving understanding of bruxism as a behavior with potential negative consequences, there is a need for objective methods to assess treatment efficacy in symptomatic patients. This study specifically targets patients presenting with masticatory muscle-related symptoms associated with bruxism behavior. The primary objective is to quantify biomechanical changes in masseter muscle properties following BoNT-A treatment, thereby providing an objective assessment tool for therapeutic interventions. This approach aligns with current recommendations for evidence-based treatment of bruxism-related symptoms while avoiding overtreatment of asymptomatic individuals.

## 2. Results

For statistical analysis, patients were divided into three groups (tertiles) based on baseline biomechanical parameters of the masseter muscles, using the 33rd and 67th percentile cut-offs. This stratification allowed assessment of BoNT-A effect relative to initial muscle tension and stiffness:

- Tertile I included patients whose baseline values were below the 33rd percentile, representing the lowest levels of masseter muscle tension and stiffness.
- Tertile II consisted of patients with baseline values between the 33rd and 67th percentiles, representing intermediate levels.
- Tertile III included patients above the 67th percentile, reflecting the highest baseline values with elevated muscle tension and stiffness.

**Masseter muscle Tension at rest**

No statistically significant changes in resting muscle tension were observed in tertiles I and II at either 3 weeks (V2) or 3 months (V3) following BoNT-A administration. In tertile III, which consisted of patients with the highest initial muscle tension, a significant decrease was observed after 3 weeks (V2) and persisted at the 3-month follow-up (V3).

**Masseter muscle Tension at maximum tension**

All three groups demonstrated a significant reduction in muscle tension during maximal biting force, as measured 3 weeks after injection (V2). At the 3-month follow-up (V3), tension levels in groups I and II had returned to values comparable to baseline. In contrast, group III maintained a moderately reduced tension level relative to the initial measurement, suggesting a potentially longer-lasting effect of the botulinum toxin.

**Masseter muscle Stiffness**

During measurements performed at rest, muscle stiffness in each tertile remained relatively stable over time. In contrast, measurements taken during muscle contraction revealed a decrease in stiffness across all groups at the 3-week mark (V2), with the most pronounced reductions observed in tertiles II and III. By the 3rd month (V3), stiffness values tended to return toward baseline—completely in group I and partially in groups II and III.

**Masseter muscle Elasticity**

As expected, masseter muscle elasticity was inversely proportional to muscle stiffness. At rest, no significant changes in elasticity were observed in any group. However, during muscle contraction, all three tertiles demonstrated an increase in elasticity at the 3-week appointment (V2). By 3 months (V3), elasticity values in tertiles II and III had returned to baseline, whereas in tertile I, they remained slightly elevated, possibly indicating a more subtle and less sustained effect of the botulinum toxin in this patient group.

**Masseter muscle Relaxation Time**

At rest, muscle relaxation time showed no significant changes, although a slight tendency toward its shortening was noted at the 3-week appointment (V2). In contrast, during measurements performed on biting muscle, all tertiles exhibited a prolongation of relaxation time at V2. By 3 months (V3), values in tertiles II and III had returned to baseline, while in tertile I, relaxation time remained moderately elevated.

**Masseter muscle Creep**

At rest, no significant differences in creep values were observed between the first and second measurements, nor between the second and third. A small but statistically significant reduction was noted only between the baseline (V1) and the 3-month visit (V3), indicating slightly lower creep values at that time point. During measurements conducted under muscle tension, creep values increased significantly at 3 weeks (V2), then declined by 3 months (V3) across all tertiles. This consistent pattern suggests that the observed changes result from the pharmacological effects of BoNT-A rather than baseline muscle characteristics.

**Results summary:**

- BoNT-A reduces muscle tension and stiffness under tension but does not significantly affect muscles at rest in most patients.
- The effects are most pronounced after three weeks and partially diminish by three months.
- The most significant and persistent changes were observed in patients with the highest muscle tension before the injection procedure (Tertile III).
- The greatest changes were observed in measurements taken under tension, which is consistent with findings from studies on healthy individuals conducted using the same device by other researchers [45].

**Summary of BoNT-A impact on biomechanical parameters of masseter muscles by patient tertiles**

To provide a more comprehensive illustration of the differences in response to BoNT-A application, a comparative analysis based on dividing patients into tertiles according to their baseline values is presented below. Each tertile represents different biomechanical characteristics of the masseter muscles before toxin administration, enabling the observation of varied reactions based on the initial functional state of the muscle system.

- Patients with the lowest baseline masseter muscle tension and stiffness.
- Tertile II grouped individuals with average parameters.
- Tertile III included patients with the highest baseline values, reflecting elevated muscle tension and stiffness.

**Tertile I**

In this group, no significant changes in masseter muscle tension, at rest, was observed either after the 3-week (V2) or 3-month (V3) appointments following BoNT-A administration. During maximum biting, a clear reduction in tension was observed after 3 weeks (V2), with values returning to levels seen on the day of administration after 3 months (V3). Changes in muscle stiffness mirrored this pattern: after an initial decrease (at V2), a return to baseline values occurred at V3. Muscle elasticity increased during the second measurement (V2), then gradually decreased, reaching values similar to the initial readings. The relaxation time after toxin administration was prolonged in the measurement taken at V2 and remained slightly elevated even after 3 months (V3), which may indicate a longer persistence of BoNT-A’s effect in this group. Regarding the creep parameter, a slight increase was observed at rest after 3 weeks (V2), accompanied by a decrease under tension after 3 months (V3), indicating a tendency to return to baseline values in the final measurement stage.

**Tertile II**

In this tertile, muscle tension at rest remained stable throughout the study period. Tension during maximum biting decreased significantly after three weeks (V2), returning to baseline values after three months (V3). Muscle stiffness under tension also experienced a pronounced reduction in the second measurement (V2), followed by an upward trend that approached the initial values at V3. Elasticity increased after 3 weeks (V2) and returned to baseline levels after 3 months (V3). Relaxation time underwent significant prolongation at V2 and returned to normal at V3. The creep parameter behaved according to the previously described pattern—a slight increase at rest after 3 weeks (V2) and a slight decrease under muscle tension, followed by returning closer to baseline values by V3.

**Tertile III**

This group demonstrated a pronounced response to the administration of botulinum toxin. Masseter muscle tension at rest significantly decreased after 3 weeks (V2) and remained reduced upon measurement after 3 months (V3). Tension during maximum biting decreased in the second measurement (V2), and after 3 months (V3), it remained lower compared to the baseline value. Muscle stiffness under tension experienced a significant reduction at V2, with a partial return at V3. Elasticity increased in the second measurement (V2), then returned to baseline values at V3. The relaxation time was prolonged significantly after 3 weeks (V2); however, after 3 months (V3), values comparable to the initial measurement were obtained. In this group, a temporary increase in the creep parameter under tension was also noted (V2), subsiding in the following measurement (V3). At rest, a small but statistically significant decrease in creep at V3 was observed.

**Interaction between Tertile and Time**

**Relaxed state measurements**

Interaction analysis (tertile × time) showed significant effects for muscle tone and stiffness at rest, indicating different trajectories across tertiles. Patients in tertile III showed more pronounced and sustained improvements compared to weaker or absent changes in tertiles I and II. Conversely, variables such as elasticity, relaxation time, and creep at rest showed no significant interaction, suggesting similar responses across groups.

**Contracted state measurements**

Statistically significant tertile × time interactions were observed for all biomechanical parameters measured during contraction. This indicates that changes over time depended on baseline muscle state. While all tertiles showed significant improvements at 3 weeks (V2), the durability of these effects varied. Tertile III exhibited the most pronounced and sustained reductions in tone and stiffness, and increases in elasticity and relaxation time, with partial improvements still evident at 3 months (V3). In contrast, tertiles I and II generally returned to baseline by the final follow-up. These findings suggest BoNT-A injections may deliver greater and longer-lasting benefits in patients with more severe initial muscle hyperactivity.

**Final Conclusions**

In Tertile I, characterized by the lowest baseline values across measured parameters, the mildest response was observed, with most values returning to initial levels within three months.

Tertile II exhibited a more pronounced response to botulinum toxin, although a tendency toward parameter normalization was observed by the third visit (V3)

In Tertile III, including individuals with the highest baseline measurements, the BoNT-A effect was both more substantial and longer-lasting, implying that the intervention may be particularly beneficial in patients with severe masticatory muscle symptoms associated with bruxism behavior and elevated baseline masseter activities.

## 3. Discussion

This study represents the first systematic attempt to objectively quantify biomechanical changes in masseter muscle properties following BoNT-A injections in patients with bruxism-related masticatory muscle symptoms. The findings should be interpreted within the contemporary understanding of bruxism as a behavior that may have both positive and negative consequences, rather than a pathological condition requiring universal treatment. This evolving perspective requires careful interpretation of correlated studies, as interventions such as BoNT-A address muscle activity but not the underlying biopsychosocial causes.

The present paper aimed to assess objective changes in masseter muscle parameters using the MyotonPro device after administration of BoNT-A. Our results indicate significant changes in resting tension, stiffness, elasticity, relaxation, and creep of the masseter muscle following BoNT-A injection, suggesting a beneficial impact on the biomechanical properties of muscles in patients with bruxism-related symptoms [46]. The psychological component has to be underlined as well. As demonstrated by recent research, emotional stress often manifests as increased muscle tension, creating a cycle of muscle fatigue and symptom development [47]. BoNT-A treatment may interrupt this cycle by reducing the peripheral muscle component, potentially providing symptomatic relief. However, addressing underlying psychological factors remains crucial for long-term management. Moreover, growing evidence indicates that the administration of BoNT to affected muscles exerts effects extending beyond the purely motor domain, influencing pain perception, mood regulation, and even cognitive function. This phenomenon, referred to as the “non-motor effect” of botulinum toxin treatment, underscores the broader therapeutic potential of BoNT and its relevance in the comprehensive management of conditions such as bruxism [26]

Observed decrease in masseter muscle resting tension after BoNT-A injection aligns with the neurotoxin’s mechanism of action, which inhibits acetylcholine release at the neuromuscular synapse level, weakening muscle contraction [48]. This effect helps reduce excessive masticatory muscle activity. The decrease in resting tension may also enhance microcirculation in the muscle, potentially alleviating pain symptoms experienced by patients [49,50].

Reduction in masseter muscle transverse stiffness, particularly pronounced during activation, indicates an increased susceptibility to stretching. From a biomechanical perspective, decreased muscle stiffness may lead to lower reaction forces transmitted to the TMJ during clenching and grinding episodes. Reduced joint loading may help decrease the risk of developing or worsening TMD, which often coexist with bruxism [51,52]. Muscle elasticity tends to behave in an opposite manner to stiffness, aligning with the physiological properties of muscle tissue [53].

A prolonged relaxation time of the masseter muscle after BoNT-A administration may indicate reduced overreactivity and a decreased tendency to maintain prolonged tension. Decreased relaxation time of masticatory muscles is frequently observed in patients with bruxism-related symptoms [53]. Our results suggest that BoNT-A may assist in normalizing this parameter. Changes in masseter muscle creep following BoNT-A application may reflect alterations in its viscoelastic properties. Increased creep may indicate a better muscle ability to adapt to prolonged loading, potentially reducing the risk of muscle fatigue and pain development [49,54]. It is important to emphasize that the most significant BoNT-A effects, measured by objective MyotonPro parameters, were observed in patients with baseline values most deviating from physiological norms. This suggests that patients with more severe bruxism symptoms may gain the greatest therapeutic benefit from BoNT-A injections. At the same time, the use of a uniform BoNT-A dose for all patients raises questions about the potential benefits of individualizing dosing based on baseline muscle parameters and symptom severity—which aligns with Jost’s suggestion regarding BoNT-A dosing for various indications [55]. Some patients reported a subjective sense of improvement following BoNT-A administration, including better sleep quality and reduced pain in the teeth, muscles, TMJ, and head—findings that may indicate a positive therapeutic effect. However, to reliably confirm and quantify these outcomes, such observations should be supported by objective assessment tools, such as the Visual Analog Scale (VAS). This aligns with the findings of systematic reviews indicating that BoNT-A can effectively reduce pain and enhance functioning in patients with bruxism [56]. However, it is important to note that not all patients, even those with objective improvements in muscle parameters, reported significant subjective improvement. In addition to biomechanical aspects, psychosocial factors must also be considered. Emotional load and stress can manifest as sustained daytime bracing, which may play a critical role in the onset of musculoskeletal symptoms. Several studies [7,57,58,59] underline the importance of including psychological variables in bruxism research.

This stem from the multifactorial etiology of bruxism, where psychological and behavioral factors play a critical role. Long-term experience with the use of botulinum toxin in neurological patients with various neuromuscular disorders indicates that, in many cases, treatment initiation is often based on an arbitrary clinical decision [60]. Current guidelines highlight that bruxism may represent a condition that clinically manifests through characteristic symptoms of masticatory muscle hyperactivity, thus therapeutic interventions should primarily aim to mitigate the negative consequences of this behavior rather than to “cure” it per se. Nevertheless, patients frequently seek relief from pain and other functional and psychosocial burdens associated with bruxism behaviors, and the clinician’s role also encompasses improving overall patient comfort and quality of life. Therefore, BoNT-A therapy can be considered as part of a multimodal management strategy including behavioral approaches.

As highlighted by Greene [19] and others, patients should be protected from unnecessary invasive interventions. Considering the ongoing controversies, it remains essential to avoid overtreatment from both ethical and clinical perspectives.

## 4. Conclusions

This study provides evidence that there are objective measurable biomechanical changes in masseter muscle properties in patients with bruxism-related masticatory muscle symptoms after BoNT-A treatment.

While objective changes in muscle parameters measured by MyotonPro may provide evidence of BoNT-A’s effect, the clinical efficacy of this treatment would be more convincingly demonstrated through randomized controlled trials (RCTs). Future researchers should prioritize the correlation between biomechanical improvements and patient-reported symptom relief, as this relationship is not fully understood. Identifying patient subgroups that may benefit most from BoNT-A treatment is an important direction for future research, which should also adopt validated assessment tools such as the STAB, stratify patients according to bruxism phenotypes (sleep vs. awake, grinding vs. bracing), and evaluate combined therapeutic strategies addressing both muscular and psychosocial aspects of this complex condition.

In conclusion, the greatest effects were observed in individuals with the highest baseline measurements, where the intervention was both more substantial and longer-lasting. These findings suggest that BoNT-A may be particularly beneficial for patients with severe masticatory muscle symptoms and elevated baseline masseter activity. Nevertheless, treatment decisions should be guided by the presence of negative consequences rather than the bruxism behavior alone.

## 5. Limitations

The findings of our study must be interpreted with caution in light of the evolving conceptualization of bruxism. As highlighted in recent consensus papers, bruxism is not a disorder but a behavior that may or may not have clinical consequences [4,44]. Our inclusion criterion of 60% positive BruxScreen items was a pragmatic choice in the absence of universally accepted diagnostic thresholds; however, we recognize that the STAB questionnaire is currently regarded as the reference standard for self-reported bruxism assessment. This limitation should be considered when interpreting the external validity of our results. Regarding the INfORM/IADR recommendations, we recognize their relevance and importance. However, these guidelines were not available at the time the study rationale was developed in 2023. Consequently, the study design and inclusion criteria were based on the most appropriate standards accessible at that time. Future research should aim to align more closely with the updated INfORM/IADR recommendations to ensure consistency with current international standards. Moreover, while some reports of symptomatic improvement were noted, systematic patient-reported outcome measures are lacking in our study. Future studies should incorporate validated assessment tools such as the Visual Analog Scale (VAS) for pain, quality of life measures, and standardized bruxism assessment protocols.

## 6. Materials and Methods

This study was conducted (November 2023–July 2024) among individuals with symptoms consistent with bruxism seeking consultation in the Temporomandibular Disorders Clinic, Poznań, Poland. In this study, “symptoms of bruxism” were operationalized as a combination of self-reported parafunctional behaviors (e.g., clenching, grinding, bracing) and positive findings on clinical examination (e.g., tooth wear facets, muscle hypertrophy, tenderness). Only individuals fulfilling both criteria were considered eligible for further screening. A total of 127 patients met these preliminary criteria and were proposed for BoNT-A injections. The study was approved by the Bioethics Committee of Poznan University of Medical Sciences (approval no. 804/22).

Qualification criteria were:

- Age 18–40 years
- Occurrence of 60% or more bruxism criteria based on the BruxScreen form (interview and clinical examination), combined with clinically significant masticatory muscle symptoms (pain, tension, fatigue) that interfere with daily function. The qualification threshold of 60% bruxism symptoms was established pragmatically, serving as a compromise between sensitivity and specificity in the absence of diagnostic criteria. While this cut-off lacks formal empirical validation, it was selected to maintain clinical relevance. Recent literature [61,62,63] underscores the methodological challenges in defining bruxism severity and highlights the need for more refined assessment tools. Future studies should incorporate standardized approaches, such as the STAB protocol, and combine validated questionnaires with instrumental or ambulatory monitoring methods where feasible. Each participant was asked to complete the Oral Behavior Checklist (OBC) [64,65]. Based on its results, individuals reporting characteristic bruxism behaviors occurring more than twice per week were selected for further screening. These participants subsequently completed the BruxScreen questionnaire, which, at the time, was recommended by a group of international experts for non-instrumental bruxism assessment [66]. Patients meeting the criteria based on this screening were evaluated further. While the STAB (Standardized Tool for the Assessment of Bruxism) protocol [6] represents the current gold standard for bruxism assessment, it was not yet fully available at the time of study initiation. Therefore, this study acknowledges the limitations of the assessment protocol used and the need for future studies to incorporate STAB methodology.

Exclusion criteria:

- tooth loss resulting in lack of one or more support zones
- recent maxillofacial trauma
- BMI > 30
- muscle relaxant use
- pregnancy
- allergy to albumins
- patients taking medications favoring interaction with the botulinum toxin, such as quinidine, calcium channel blockers
- parallel temporomandibular disorder (TMD) treatment according to INfORM/IADR guidelines
- significant psychological disorders requiring active treatment (as these may influence muscle tension patterns independently)

Two individuals were excluded from the study post-BoNT-A administration: one initiated masticatory muscle physiotherapy, and the other presented with trauma-related complications stemming from sports activity.

**Administration Technique:**

The analysis of medical records revealed that all patients were informed about the possible side effects of BoNT-A injections, and all provided written informed consent before procedure initiation. The 100 units of lyophilized BoNT-A (Botox, Allergan, Inc., Irvine, CA, USA) were diluted with 2.5 mL of 0.9% sodium chloride according to manufacturer recommendations for concentration within facial muscles [67], for a dose of 4.0 U/0.1 mL. All administrations were performed under ultrasound control using a 20 mm 30G needle. During administration, the patient was in a supine position. Additionally, the posterior, superior, and inferior borders of the masseter muscle were identified and marked on the skin prior to injection, which was performed under ultrasound guidance. The needle was positioned perpendicular to the skin and inserted into the appropriate muscle layer. After a negative aspiration test, the prepared solution was gradually deposited to minimize the patient’s feeling of distension and damage caused by the introduction of volume, thereby reducing the risk of potential spread to adjacent anatomical areas. All injections were administered by the same person to ensure consistency. Each patient received a total of 80 units BoNT-A at 3 injection points per masticatory muscle

A standardized dose of 80 units of BoNT-A falls within the reference range commonly used in the management of bruxism, with the literature reporting effective doses between 50 and 100 units per patient [42,68,69].

Using a fixed dose of 80 units is methodologically sound, as it reduces variability related to individualized dosing and allows for a more objective evaluation of intervention outcomes. Moreover, there are currently no validated protocols for adjusting BoNT-A doses based on ultrasound muscle parameters [70] or number of injection sites, which further justifies the use of a standardized reference dose in this study.

- The first injection (15 U) was administered in the deep belly of the muscle, near the mandibular angle 1.5 cm from mandibular ramus and 1.5 cm from mandibular body.
- The second injection (15 U) was administered in the superficial belly of the muscle, approximately 0.5 cm below its point of greatest prominence.
- The third injection (10 U) was administered in the superficial belly, 1.5 cm above muscle greatest prominence.

No complications or side effects were observed in patients who received injections.

The final masseter biomechanical properties study group consisted of 57 participants (29 female and 28 male). The exclusion criterion of BMI > 30 was established based on documented limitations in the accuracy of myotonometric measurements in individuals with excessive adipose tissue. Experimental studies have shown that when the thickness of the subcutaneous fat layer exceeds 0.689 ± 0.035 cm, MYOTON measurements become unreliable and fail to detect true changes in the mechanical properties of muscle tissue [71].

To assess muscle properties such as tone, stiffness, elasticity, relaxation and creep [72], the MyotonPro device (MyotonPRO, Myoton AS., Tallinn, Estonia) was used. MyotonPRO offers a unique, accurate, and sensitive method for the objective examination of superficial skeletal muscles [73]. Measurement using the device involves non-invasive examination where a gentle mechanical impulse (0.4 N) is applied to the skin, allowing assessment of superficial tissue deformation, detection of natural soft tissue vibration damping, and measurement of the vibration frequency of the tested element [74].

Measured values:

MyotonPro device was used to measure five parameters. They are described by the producent as follows [75]:

- Tone is characterized by Oscillation frequency [Hz] (muscle stiffness) of biological soft tissues on the cellular level. Oscillation Frequency characterizes the tone of superficial skeletal muscles in their passive or resting state without voluntary contraction (EMG signal silent). The Oscillation Frequency of a muscle in its contracted state characterizes state of tension.
- Dynamic Stiffness [N/m] characterizes the resistance of biological soft tissues to a force of deformation. The term Dynamic Stiffness originates from the dynamic measurement method applied in Myoton technology. The inverse of stiffness is compliance.
- Logarithmic Decrement [arb] characterizes the dampening of tissue oscillation. The faster the tissue oscillation fades, the higher the dissipation of mechanical energy induced by the measurement impulse. The decrement in tissues’ natural oscillation inversely describes elasticity. Elasticity is the biomechanical property of soft tissues that characterizes the ability to recover its initial shape from being deformed. The higher the decrement, the lower the elasticity. In theory, a decrement of zero (0) represents absolute elasticity (absence of dampening). The inverse of elasticity is plasticity.
- Mechanical Stress Relaxation Time [ms] characterizes tissue’s recovery time from displacement. The higher a tissue’s tension or stiffness, the faster a tissue recovers its shape, meaning the lower the value.
- Ratio of Relaxation and Deformation time [arb] characterizes creep, the gradual elongation of tissue over time when placed under constant tensile stress. The higher a tissue’s tension, structural integrity, or stiffness, the higher its resistance to creep, meaning the lower the value.

Measurement Procedure:

The patient was asked to take a seat in the dental chair, which was adjusted to a 75-degree angle relative to the seat. This position was saved in the settings memory to ensure consistency in positioning for every patient examined.

The examiner then determined the most significant prominence of the masseter muscle for the right and left sides. The patient was instructed to bite on a modeling wax plate, thus inducing masseter muscle contraction. The most significant prominence was palpated and marked with a dot on the skin using the marker included with the Myoton device. Location of the most significant prominence was confirmed using ultrasound (Hitachi ALOKA F37, Mitaka, Japan) [76,77].

**Figure 1:**
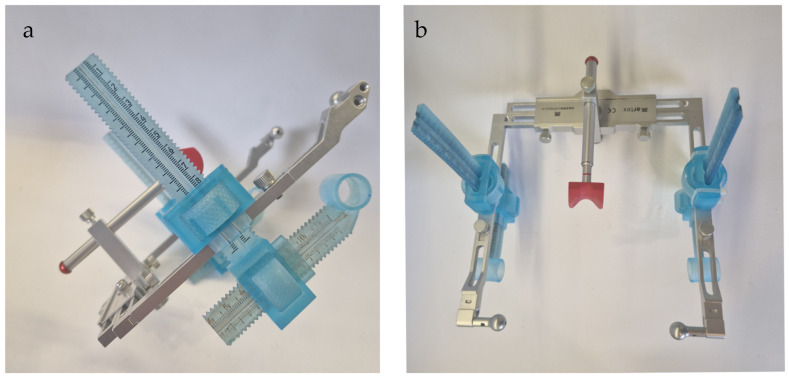
Amann Girrbach face bow with 3D-printed extensions allowing for the reproducible localization of the most prominent point of the masseter muscle. (**a**) From the side, (**b**) from the top.

**Figure 2:**
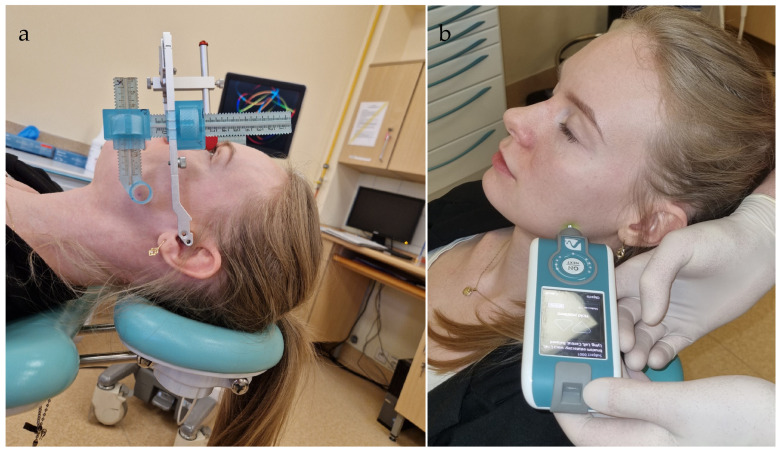
(**a**) Face-bow mounted on a patient. (**b**) Measurement of the parameters using MyotonPRO.

To ensure consistent probe placement across multiple visits (a critical requirement in longitudinal myotonometric studies), a custom device based on the Amann Girrbach face bow (Maeder, Austria) (Figure 1 and Figure 2a) was used. This system enabled accurate registration and documentation of spatial coordinates, ensuring the measurement probe could be repositioned at identical anatomical sites and minimizing errors caused by even slight variations in probe location. Once the coordinates were recorded, the face bow was removed from the patient’s head.

The next stage involved measuring masseter muscle parameters using the Myoton device (Tallinn, Estonia) (Figure 2b) according to the examination technique provided by the device manufacturer. Each measurement cycle consisted of the following elements:

- Left masseter muscle examination at rest (5 measurements).
- Right masseter muscle examination at rest (5 measurements).
- Left masseter muscle examination at maximum tension (5 measurements).
- Right masseter muscle examination at maximum tension (5 measurements).

Muscle tension was achieved by instructing the patient to bite on a 2 mm modeling wax plate. A total of 20 measurements of masseter muscle parameters were performed in a single session.

The entire research cycle was based on performing the measurements as mentioned above in the following stages:

- Visit 1 (V1)—examination before BoNT-A administration (baseline).
- Visit 2 (V2)—examination 3 weeks after BoNT-A administration.
- Visit 3 (V3)—examination 3 months after BoNT-A administration.

The timing of follow-up assessments (3 weeks and 3 months) was selected based on the pharmacodynamics of BoNT-A. Botulinum toxin reaches its peak effect approximately 10 days after injection, with the full therapeutic effect stabilizing within 2–3 weeks. This supports the first follow-up visit at 3 weeks as the optimal point for evaluating the maximum therapeutic effect [78]. The 3-month (12-week) follow-up aligns with the established standard interval between BoNT-A injections [79] and corresponds to the period when the therapeutic effect typically begins to wane, allowing for an assessment of intervention durability prior to planning a potential reinjection [80]. Three months are further supported by pharmacological data indicating that the therapeutic effects of BoNT-A typically last between 8 and 12 weeks [70].

Visit V3 was permitted to be delayed by up to 7 days in cases of holidays or patient-related personal or health issues.

Data from individual measurements were recorded, the mean value was calculated for each condition tone, stiffness, elasticity, relaxation and creep, and all results were subjected to statistical analysis.

**Statistical analysis**

Data normality was assessed using the Shapiro–Wilk test. Participants were divided into tertiles by splitting the distribution of the relevant continuous variable at the 33rd and 67th percentiles. To evaluate changes in variables over time (V1, V2, V3), and to assess whether these changes differed across tertiles, a repeated measures analysis of variance (mixed between–within ANOVA) was performed. The factor variable (three levels—tertiles) was treated as a between-subjects factor, while time was included as a within-subjects factor. The model also included an interaction term between tertile group and time. Individual participants were nested within tertile groups, allowing for control of inter-individual variability within groups. The residual error term accounted for within-subject variability across repeated measures (Table 1 and Table 2).

When analyzing the effect of time, the assumption of sphericity was tested using Mauchly’s test. In cases where this assumption was violated, the Huynh–Feldt correction was applied to adjust the degrees of freedom for the within-subject effects.

It is important to note that the tertile grouping variable was derived by stratifying participants based on their baseline (V1) values of the dependent variable. Therefore, differences between tertiles at baseline are inherently defined by the group construction and were not the result of independent random assignment or experimental manipulation. Consequently, the main effect of the tertile factor was not interpreted, as it merely reflects the predefined structure of the data and does not provide meaningful insight into between-group differences. Instead, the analysis focused on the interaction between tertile group and time, which allows for the evaluation of whether trajectories of change differ depending on baseline levels.

When significant main effects or interactions were found, pairwise post hoc comparisons between time points within each tertile were conducted using Tukey’s test to adjust for multiple comparisons.

All tests were two-tailed, and statistical significance was set at *p* < 0.05. All analyses were performed using Stata version 18 (StataCorp LLC, College Station, TX, USA)

## Figures and Tables

**Table 1:** Results of repeated measures analysis: time effects across V1, V2, and V3, and Tertile × Time interaction.

Site	Muscle State	Variable	Interaction *Tertile* × *Time* (*p* < 0.05)	Tertile I (n = 19)			Tertile II (n = 19)			Tertile III (n = 19)		
				V1 vs. V2	V1 vs. V3	V2 vs. V3	V1 vs. V2	V1 vs. V3	V2 vs. V3	V1 vs. V2	V1 vs. V3	V2 vs. V3
Left masseter muscle	Relaxed	Tone	F(4.108) = 3.80 *p* = 0.007	# F(2,36) = 1.98 *p* = 0.153			# F(2,36) = 1.40 *p* = 0.260			# F(2,36) = 11.30 *p* < 0.001		
										<0.001 *	0.019 *	0.161 *
										1 > (2 = 3)		
		Stiffness	F(4.108) = 2.66 *p* = 0.036	# F(2,36) = 8.37 *p* = 0.001			# F(2,36) = 5.73 *p* = 0.0067			# F(2,36) = 1.36 *p* = 0.270		
				0.003 *	0.003 *	1.000 *	0.608 *	0.006 *	0.063 *			
				1 < (2 = 3)			1 < 3					
		Elasticity	F(4.108) = 1.35 *p* = 0.255	# F(2,36) = 2.61 *p* = 0.087			# F(2,36) = 0.81 *p* = 0.453			# F(2,36) = 4.13 *p* = 0.024		
										0.904 *	0.077 *	0.029 *
										2 > 3		
		Relaxation	F(4.108) = 1.43 *p* = 0.229	# F(2,36) = 2.39 *p* = 0.106			# F(2,36) = 3.65 *p* = 0.036			# F(2,36) = 10.77 *p* < 0.001		
							0.355 *	0.027 *	0.398 *	0.048 *	<0.001 *	0.092 *
							1 < 3			1 > (2 = 3)		
		Creep	F(4.108) = 1.52 *p* = 0.202	# F(2,36) = 2.60 *p* = 0.216			# F(2,36) = 7.07 *p* = 0.003			# F(2,36) = 9.98 *p* < 0.001		
							0.520 *	0.002 *	0.039 *	0.796 *	0.001 *	0.003 *
							(1 = 2) > 3			(1 = 2) > 3		
	Contracted	Tone	F(4.108) = 19.98 *p* < 0.001	# F(2,36) = 24.07 *p* < 0.001			# F(2,36) = 67.57 *p* < 0.001			# F(2,36) = 259.57 *p* < 0.001		
				<0.001 *	0.226 *	<0.001 *	<0.001 *	<0.001 *	<0.001 *	<0.001 *	<0.001 *	<0.001 *
				2 < (1 = 3)			2 < 3 < 1			2 < 3 < 1		
		Stiffness	F(4.108) = 24.07 *p* < 0.001	# F(2,36) = 19.97 *p* < 0.001			# F(2,36) = 61.64 *p* < 0.001			# F(2,36) = 235.62 *p* < 0.001		
				<0.001 *	0.043 *	<0.001 *	<0.001 *	<0.001 *	<0.001 *	<0.001 *	<0.001 *	<0.001 *
				2 < 1 < 3			2 < 3 < 1			2 < 3 < 1		
		Elasticity	F(4.108) = 6.64 *p* < 0.001	# F(2,36) = 66.20 *p* < 0.001			# F(2,36) = 64.76 *p* < 0.001			# F(2,36) = 19.68 *p* < 0.001		
				<0.001 *	0.001 *	<0.001 *	<0.001 *	0.332 *	<0.001 *	0.001 *	0.073 *	<0.001 *
				1 < 3 < 2			(1 = 3) < 2			(1 = 3) < 2		
		Relaxation	F(4.108) = 142.27 *p* < 0.001	# F(2,36) = 142.27 *p* < 0.001			# F(2,36) = 41.05 *p* < 0.001			# F(2,36) = 21.43 *p* < 0.001		
				<0.001 *	<0.001 *	<0.001 *	<0.001 *	0.463 *	<0.001 *	<0.001 *	0.512 *	<0.001 *
				1 < 3 < 2			(1 = 3) < 2			(1 = 3) < 2		
		Creep	F(4.108) = 4.02 *p* = 0.004	# F(2,36) = 172.49 *p* < 0.001			# F(2,36) = 37.12 *p* < 0.001			# F(2,36) = 32.91 *p* < 0.001		
				<0.001 *	<0.001 *	<0.001 *	<0.001 *	0.238 *	<0.001 *	<0.001 *	0.494 *	<0.001 *
				1 < 3 < 2			(1 = 3) < 2			(1 = 3) < 2		
Right masseter muscle	Relaxed	Tone	F(4.108) = 4.55 *p* = 0.002	# F(2,36) = 1.19 *p* = 0.316			# F(2,36) = 0.38 *p* = 0.685			# F(2,36) = 14.40 *p* < 0.001		
										<0.001 *	0.001 *	0.581 *
										(2 = 3) < 1		
		Stiffness	F(4.108) = 0.84 *p* = 0.504	# F(2,36) = 2.17 *p* = 0.129			# F(2,36) = 1.58 *p* = 0.221			# F(2,36) = 1.74 *p* = 0.190		
		Elasticity	F(4.108) = 2.05 *p* = 0.092	# F(2,36) = 0.25 *p* = 0.778			# F(2,36) = 3.05 *p* = 0.061			# F(2,36) = 3.86 *p* = 0.030		
										0.932 *	0.038 *	0.083 *
										3 < 1		
		Relaxation	F(4.108) = 1.95 *p* = 0.107	# F(2,36) = 0.45 *p* = 0.642			# F(2,36) = 3.58 *p* = 0.040			# F(2,36) = 7.01 *p* = 0.003		
							0.994 *	0.076 *	0.061 *	0.559 *	0.002 *	0.035 *
							-			(1 = 2) > 3		
		Creep	F(4.108) = 1.16 *p* = 0.331	# F(2,36) = 0.80 *p* = 0.458			# F(2,36) = 2.60 *p* = 0.088			# F(2,36) = 5.91 *p* = 0.006		
										0.346 *	0.004 *	0.125 *
										1 > 3		
	Contracted	Tone	F(4.108) = 25.23 *p* < 0.001	# F(2,36) = 49.82 *p* < 0.001			# F(2,36) = 111.97 *p* < 0.001			# F(2,36) = 150.94 *p* < 0.001		
				<0.001 *	0.935 *	<0.001 *	<0.001 *	<0.001 *	<0.001 *	<0.001 *	<0.001 *	<0.001 *
				2 < (1 = 3)			2 < 3 < 1			2 < 3 < 1		
		Stiffness	F(4.108) = 19.11 *p* < 0.001	# F(2,36) = 29.17 *p* < 0.001			# F(2,36) = 75.94 *p* < 0.001			# F(2,36) = 120.47 *p* < 0.001		
				<0.001 *	0.637 *	<0.001 *	<0.001 *	<0.001 *	<0.001 *	<0.001 *	<0.001 *	<0.001 *
				2 < (1 = 3)			2 < 3 < 1			2 < 3 < 1		
		Elasticity	F(4.108) = 8.37 *p* < 0.001	# F(2,36) = 88.55 *p* < 0.001			# F(2,36) = 17.55 *p* < 0.001			# F(2,36) = 14.27 *p* < 0.001		
				<0.001 *	0.001 *	<0.001 *	<0.001 *	0.399 *	<0.001 *	0.287 *	0.002 *	<0.001 *
				1 < 3 < 2			(1 = 3) < 2			3 < (1 = 2)		
		Relaxation	F(4.108) = 9.06 *p* < 0.001	# F(2,36) = 109.85 *p* < 0.001			# F(2,36) = 94.39 *p* < 0.001			# F(2,36) = 37.51 *p* < 0.001		
				<0.001 *	<0.001 *	<0.001 *	<0.001 *	0.023 *	<0.001 *	<0.001 *	0.176 *	<0.001 *
				1 < 3 < 2			1 < 3 < 2			(1 = 3) < 2		
		Creep	F(4.108) = 9.68 *p* < 0.001	# F(2,36) = 118.70 *p* < 0.001			# F(2,36) = 70.22 *p* < 0.001			# F(2,36) = 63.12 *p* < 0.001		
				<0.001 *	<0.001 *	<0.001 *	<0.001 *	0.238 *	<0.001 *	<0.001 *	0.036 *	<0.001 *
				1 < 3 < 2			1 < 3 < 2			3 < 1 < 2		

#—Indicates results from repeated measures ANOVA. *—Indicates *p* values obtained from Tukey-adjusted post hoc tests.

**Table 2:** Mean measured values and standard deviation across times and tertiles statistics.

Site	Muscle State	Variable	Tertile I (n = 19)			Tertile II (n = 19)			Tertile III (n = 19)		
			Mean Value			Mean Value			Mean Value		
			±Standard Deviation			±Standard Deviation			±Standard Deviation		
			V1	V2	V3	V1	V2	V3	V1	V2	V3
left masseter muscle	relaxed	Tone	13.4	13.6	13.9	15.0	15.2	15.7	17.0	15.8	16.3
			±0.9	±1.4	±1.6	±0.3	±1.1	±2.2	±1.6	±1.2	±1.5
		Stiffness	239	275	275	288	304	343	348	340	357
			±17	±46	±47	±9	±42	±83	±42	±42	±45
		Elasticity	1.64	1.72	1.69	1.82	1.87	1.80	2.10	2.12	2.00
			±0.07	±0.13	±0.21	±0.05	±0.16	±0.26	±0.18	±0.23	±0.20
		Relaxation	16.3	16.6	15.1	19.0	18.4	17.8	22.0	20.5	19.3
			±1.9	±2.0	±3.1	±0.4	±1.7	±2.4	±2.6	±3.3	±2.9
		Creep	1.02	1.15	1.01	1.17	1.12	1.01	1.37	1.34	1.18
			±0.09	±0.50	±0.22	±0.03	±0.11	±0.19	±0.19	±0.19	±0.20
	contracted	Tone	19.4	16.1	20.7	23.1	16.1	20.3	26.7	16.8	21.3
			±1.5	±1.8	±3.3	±0.9	±1.9	±2.8	±1.5	±1.3	±2.1
		Stiffness	463	331	539	622	348	512	786	346	570
			±61	±56	±157	±57	±76	±137	±50	±44	±104
		Elasticity	0.83	1.50	1.08	1.07	1.58	1.14	1.37	1.68	1.19
			±0.10	±0.16	±0.23	±0.07	±0.21	±0.20	±0.15	±0.30	±0.23
		Relaxation	6.5	14.1	8.8	8.9	15.8	9.9	11.4	15.6	10.4
			±0.6	±2.2	±1.4	±0.7	±3.7	±2.6	±1.5	±2.9	±3.1
		Creep	0.44	0.90	0.58	0.57	0.91	0.64	0.70	0.98	0.65
			±0.03	±0.11	±0.11	±0.03	±0.20	±0.13	±0.09	±0.13	±0.18
right masseter muscle	relaxed	Tone	13.2	13.3	13.7	14.9	14.7	14.7	16.6	15.2	15.5
			±0.8	±1.1	±1.9	±0.3	±0.8	±1.6	±1.4	±1.2	±1.6
		Stiffness	248	255	268	283	292	307	339	323	342
			±18	±34	±47	±9	±37	±64	±38	±38	±61
		Elasticity	1.69	1.70	1.73	1.86	1.87	1.76	2.10	2.08	1.94
			±0.09	±0.18	±0.29	±0.03	±0.17	±0.20	±0.15	±0.23	±0.30
		Relaxation	17.1	17.7	17.3	19.2	19.2	17.7	22.2	21.6	20.1
			±1.5	±2.5	±3.0	±0.5	±1.5	±2.9	±2.0	±2.9	±3.0
		Creep	1.06	1.10	1.05	1.17	1.18	1.09	1.36	1.31	1.25
			±0.09	±0.16	±0.17	±0.02	±0.10	±0.18	±0.14	±0.17	±0.14
	contracted	Tone	19.7	15.5	19.8	23.6	16.2	19.8	27.5	16.9	21.0
			±2.0	±1.6	±2.5	±0.7	±1.4	±2.2	±2.3	±1.6	±2.4
		Stiffness	467	313	490	638	348	514	791	350	552
			±93	±48	±117	±23	±59	±116	±78	±63	±119
		Elasticity	0.82	1.44	1.06	1.08	1.58	1.20	1.71	1.89	1.27
			±0.09	±0.20	±0.11	±0.12	±0.35	±0.27	±0.50	±0.28	±0.20
		Relaxation	6.5	15.4	9.5	8.3	15.2	9.8	11.8	16.1	10.6
			±0.8	±2.7	±2.2	±0.5	±2.0	±2.0	±1.9	±3.7	±2.1
		Creep	0.44	0.93	0.61	0.54	0.91	0.63	0.73	1.02	0.64
			±0.04	±0.14	±0.16	±0.03	±0.12	±0.13	±0.10	±0.17	±0.14

## Data Availability

The original contributions presented in this study are included in the article. Further inquiries can be directed to the corresponding author(s).
